# Involvement of the BDNF–TrkB–KCC2 pathway in neuropathic pain after brachial plexus avulsion

**DOI:** 10.1002/brb3.2464

**Published:** 2022-02-01

**Authors:** Shuo Zhao, Fengyu Wang, Li Wang, Yali Xu, Li Lv, Wenxu Duan, Runze Bai, Zhao Meng, Xinzhong Shao

**Affiliations:** ^1^ Department of Hand Surgery The Third Hospital of Hebei Medical University Shijiazhuang China; ^2^ Orthopaedic Department Children's Hospital of Hebei Province Shijiazhuang China

**Keywords:** BDNF, brachial plexus avulsion, K252a, KCC2, neuropathic pain, TrkB

## Abstract

**Introduction:**

Brachial plexus avulsion significantly increased brain‐derived neurotrophic factor (BDNF) release in the spinal cord. Here we investigated the involvement of the BDNF–TrkB–KCC2 pathway in neuropathic pain caused by BPA injury. We hypothesized that activation of BDNF–TrkB may inhibit neuronal excitability by downregulating KCC2 to maintain a high intracellular Cl‐concentration. We established a neuropathic pain rat model by avulsion of the lower trunk brachial plexus, and investigated the effects of the TrkB‐specific antibody K‐252a on the expression of BDNF, TrkB, and KCC2.

**Methods:**

We randomly divided 40 male SD rats into four groups. In the brachial plexus avulsion group, C8‐T1 roots were avulsed from the spinal cord at the lower trunk level. In the K252a group, 5uL K252a was applied intrathecally daily for three days after avulsion. In the sham surgery group, expose only and without damage. The control group did not undergo any treatment. Mechanical hyperalgesia and cold allodynia were analyzed by electronic pain measuring instrument and acetone spray method at different time points on days 1, 3, 7, 10, 14, and 21 after surgery. At 21 days after surgery, the expression of BDNF and TrkB in dorsal horn neurons and GFAP in astrocytes were detected by immunohistochemistry at the C5‐T1 segment of the spinal cord. The expression levels of BDNF, TrkB, and KCC2 in the C5‐T1 spinal cord were measured by Western Blot at 7 and 21 days.

**Results:**

Mechanical hyperalgesia and cold allodynia were significantly reduced in the K252a group compared with the brachial plexus avulsion group. Compared with the BPA group, BDNF, TrkB and GFAP were significantly decreased in the K252a group at 21 days after treatment by immunohistochemical test. In the WB test, the expressions of BDNF and TrkB in the K252a group were quantitatively detected to be decreased, while the expression of KCC2 was increased, which was obvious at 7 and 21 days.

**Conclusion:**

BDNF‐TrkB‐KCC2 pathway can significantly relieve neuropathic pain after BPA, and is a potential target for the treatment of neuropathic pain.

## Graphical abstract

We confirmed that K252a treatment significantly reduced the expression of brain‐derived neurotrophic factor and tyrosine kinase B, increased the expression of potassium chloride co‐transporter 2, and significantly alleviated neuropathic pain after brachial plexus avulsion (BPA). This pathway was thus an effective target for the treatment of neuropathic pain after BPA.

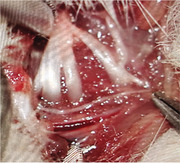

## 1 INTRODUCTION

Neuropathic pain, induced by inflammation, trauma, or drug injury of the peripheral or central nervous system, is a fast, strong, and long‐lasting pain syndrome. It is usually characterized by hyperalgesia, spontaneous pain, and ectopic pain, which typically affects the injured regions (Baron et al., 2010; Cohen & Mao, 2014). Clinically, the incidence of neuropathic pain in patients with chronic pain is approximately 6.7% (van Hecke et al., 2014). Treatment by drugs and surgery demonstrates poor efficiency with neuropathic pain, because the underlying mechanism is not fully understood (Breivik et al., 2006).

Central sensitization plays an important role in the development of neuropathic pain. A number of reports have mainly focused on the central sensitization to identify potential pathways or targets to block pain transmission, which including nerve growth factor (TGF), tumor necrosis factor (TNF), neuro‐nutrients (NT‐4), glial‐derived neurotrophic factor (GDNF), brain‐derived neurotrophic factor (BDNF), (Maisonpierre et al., 1991) as well as substance P and other small molecular peptides. Despite some progress in this field, the exact mechanism of pain transmission in central sensitization remains unclear.

BDNF is a soluble polypeptide, first isolated from pig brain extract by Barde et al. (1982), and consists of 119 amino acid residues. It is synthesized by the small‐ and medium‐diameter dorsal root ganglion neurons and is transported anterogradely to the primary afferent fibers in superficial layers I and II of the dorsal horn of the spinal cord. BDNF has two receptor types: the low‐affinity receptor p75 and the high‐affinity Trk receptor family. No biological function has been associated with binding of BDNF to p75, however, binding of BDNF to a Trk receptor, particularly its high‐affinity binding to subtype tyrosine kinase B (TrkB), plays a significant role in synaptic plasticity, and glial cell proliferation (Sasi et al., 2017). The fact that BDNF binding to TrkB leads to more phosphorylation of TrkB has been confirmed in animal models of neuropathic pain (Quintão et al., 2008), but the precise pathway remains unclear.

Potassium chloride co‐transporter 2 (KCC2) is a major Cl^−^ extruder in the central nervous system and plays a major role in Cl^−^ homeostasis (Coull et al., 2005). Shi Wenhui et al. (Shi W et al., 2015) found that the BDNF–TrkB–KCC2 pathway is involved in nicotine‐withdrawal‐induced anaphylactic pain. In animal models of inflammatory pain, neuropathic pain, and opioid‐induced hyperalgesia (Liu et al., 2017; Yajima et al., 2005) BDNF has been shown to downregulate KCC2 expression by activating the TrkB receptor.

We speculated that the BDNF/TrkB/KCC2 signaling pathway plays a role in neuropathic pain after brachial plexus avulsion (BPA). In this study, the effect of the BDNF/TrkB/KCC2 pathway on neuropathic pain was investigated in a rat model of BPA, by intrathecal application of the TrkB antagonist, K252a.

## 2 MATERIALS AND METHODS

### 2.1 Animals

In this study, 40 male Sprague–Dawley rats weighing 200−260 g were used (Hou & Xu, 2018; Wang et al., 2015). Rats were housed under mild temperature and humidity, and had free access to fresh food and water under a 12 h/12 h light/dark cycle. Efforts were made to use the minimum sample size for all experiments. All experimental procedures were approved by the Ethics Committee of the Third Hospital of Hebei Medical University.

### 2.2 Neuropathic pain model

Rats were divided into four groups: the BPA group ( *n* = 10), with lower trunk avulsion of the brachial plexus nerve; the K252a‐treatment group (K252a group; *n* = 10), with avulsion of the lower brachial plexus and intrathecal administration of K252a once a day for 3 consecutive days after surgery; the control group (*n* = 10), with no surgery or treatment; and the sham surgery group, with only exposure and separation of the brachial plexus nerves without injury.

**FIGURE 1:**
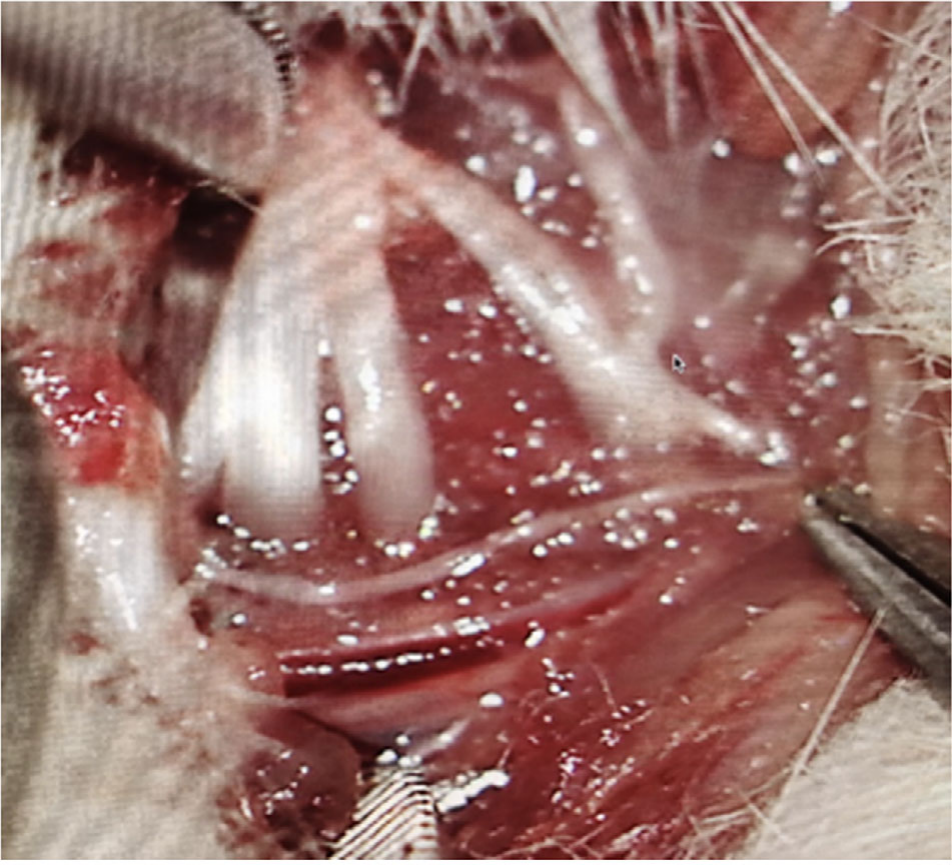
The brachial plexus of rat

**FIGURE 2:**
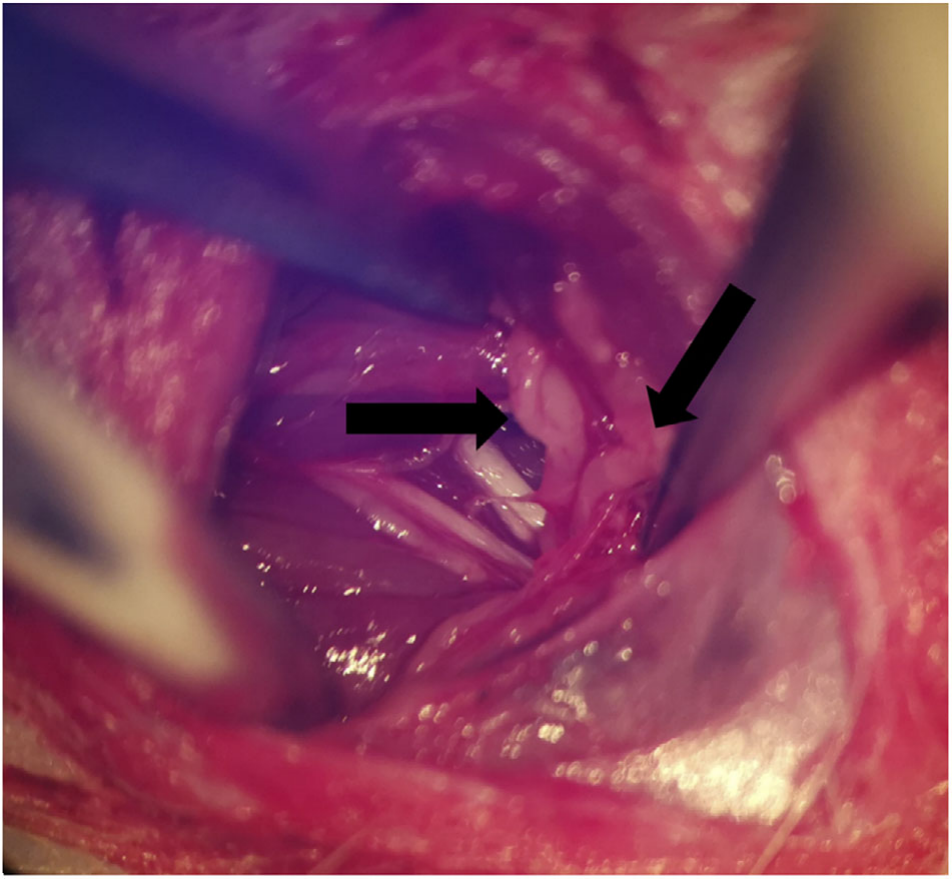
The lower trunk of brachial plexus avulsion in surgery. The black arrow shows the dorsal root ganglion

For surgery, rats were anesthetized with 1% phenobarbital (50 mg/kg).The brachial plexus was exposed through a parallel clavicular incision from the sternum to the axillary region. The pectoralis major muscle was removed, and the cephalic vein was kept intact. The subclavian blood vessels were located, and the lower trunk was separated (Figure 1). In the BPA and K252a‐treatment groups, the lower trunk was grasped with forceps and retracted from the root of the spinal cord. During the surgery, observation of cerebrospinal fluid leakage and of the dorsal root ganglion (Figure 2) was confirmed as sign of avulsion of the root (Yoon et al., 1994).

### 2.3 Paw‐withdrawal threshold for mechanical stimuli

The von Frey filament method of Rodrigues‐Filho et al. (2003) was used to induce pain, and the paw‐withdrawal threshold (PWT) was measured for the right hind paw. A 30‐cm‐high metal grid (0.8 × 0.8 cm^2^ lattice) was placed on a table, and the rats were placed on this grid and covered with a transparent glass box. Before the test, the rats were familiarized with the environment for approximately 15 min or until exploration behavior ceased. The tip of an electronic pain meter (Bioseb, Vitrolles, France) was used to stimulate the mid‐metatarsal surface of the rat's hind paw vertically through the grid, with gradually increasing intensity, until the rat suddenly lifted the paw. The point where the rat lifted the paw off the metal grid or lifted and licked the hind paw was recorded as the threshold value. Five measurements were performed at an interval of 30 s, and the average value of the five measurements was recorded. Mechanical pain thresholds at 0 days (preoperatively) and at 1, 4, 7, 10, 14, and 21 days postoperatively were recorded.

### 2.4 Cold allodynia

To evaluate cold allodynia, the acetone spray method described by Choi et al. (Yoon Choi, 1994) was used. The same setup as for the paw withdrawal test was used. After the rats were familiarized with the environment, as above, 250 μl of acetone (5°C) was sprayed onto the mid‐metatarsal surface of a hind paw. The paw lifting response of rats was scored on a scale of 0–3 points: no response, 0 points (no paw movement); mild reaction, 1 point (paw slightly lifted off the table or merely counteracting its gravity); moderate reaction, 2 points (paw is raised and suspended without contacting any surface); strong reaction, 3 points (licking, biting, or shaking the paw).

### 2.5 Drugs

The TrkB‐specific antibody K252a (Sigma, St Louis, MO) was used in the K252a‐treatment group. K252a is a potential Trk‐dependent protein tyrosine kinase inhibitor. K252a (6 μl) was injected intrathecally 30 min before the operation, and then once a day for 3 consecutive days after the operation (Buck & Winter, 1996). The injections were performed by holding the rat securely in one hand and inserting a 25 μl Hamilton syringe, into the tissues between the dorsal segments of C8 and T1, perpendicular to the vertebral column.

### 2.6 Immunohistochemistry and western blot analysis

One hour after the last behavioral test at two time points (7 days and 21 days), five rats per group selected were anesthetized intraperitoneally with phenobarbital sodium (100 mg/kg) . Cardiac perfusion was performed with 0.1 M phosphate buffer, followed by fixation with 4% paraformaldehyde. The C5–T1 sections of the spinal cord were removed from each rat. At 7 and 21 days after surgery, five rats fresh tissues from each group were selected for western blot analysis, and the remaining tissues were prepared for immunohistochemical detection of GFAP/BDNF/TrkB expression.

For immunohistochemistry, the regions analyzed were superficial laminae in the dorsal horn ipsilaterally. The number of spinal cord sections analyzed per rat was 25, the number of spinal cord sections analyzed per spinal cord segmental level was 5, the segments analyzed were the whole C5 to T1 region. The tissue sections (5 μm)were dewaxed and hydrated with xylene and gradient alcohol, washed with phosphate‐buffered saline (PBS) for 5 min, incubated with 0.3% H_2_O_2_ solution in deionized water for 30 min, and washed with PBS solution for 5 min. The tissues were then incubated in diluted normal serum at room temperature for 20 min. After pouring off the excess fluid, the tissues were incubated in the primary antibody (BDNF antibody, dilution 1:200, ProteinTech Group, Chicago, IL, USA; GFAP antibody, dilution 1:100, ProteinTech Group, Chicago, IL, USA; TrkB antibody, dilution 1:100, ProteinTech Group, Chicago, IL, USA) diluted in PBS solution for 60 min, the sections were rinsed with buffer solution for 5 min. They were then incubated with the biotin‐labeled secondary antibody (PK‐4001, dilution 1:200, Vector, Burlingame, CA) for 30 min, and then rinsed again with buffer solution for 5 min. The tissues were subsequently incubated with the VECTASTAIN ABC (PK‐4001, Vector, Burlingame, CA) reagent for 30 min, and the sections again rinsed with buffer solution for 5 min thereafter. Next, the tissue was incubated in a DAB solution of peroxidase substrate until the required staining intensity was achieved. Sections were then rinsed under running water. After dehydration in a graded series of alcohol, they were rendered transparent, and sealed (Iwasaki et al., 2013). The sections were examined under 400× magnification, and five high‐power fields were randomly selected from each section. Image Pro‐Plus software was used for image analysis and quantitative characterization. The mean integral optical density was used to evaluate the degree of section staining.

Samples for western blotting were incubated in RIPA lysis buffer containing protease inhibitor (P0013E; Biyuntian Biotechnology, Shanghai, China). The homogenate was then centrifuged at 12,000 × *g* at 4°C for 10 min, and the supernatant was collected. The protein concentration in the supernatant was then measured by BCA analysis. For each sample, the same amount of total protein was mixed with double the protein loading buffer, centrifuged at 100°C for 5 min, and then separated by SDS‐PAGE. The separated proteins were then transferred to a nitrocellulose membrane (Pall Corporation, Shanghai, China; T22740) by electrophoretic transfer. The membrane was blocked with 5% skimmed milk dissolved in PBS, at room temperature for 2 h. The membrane was incubated overnight with primary antibodies against BDNF (BDNF antibody, dilution 1:600, ProteinTech Group, Chicago, IL, USA) , TrkB (TrkB antibody, dilution 1:600, ProteinTech Group, Chicago, IL, USA) , and KCC2 (KCC2 antibody, dilution 1:600, ProteinTech Group, Chicago, IL, USA , at 4°C. After washing three times in PBS solution for 10 min each time, membranes were incubated with the corresponding secondary antibodies (Goat anti rabbit antibody Zsbio, Beijing, China) labeled with horseradish peroxidase at 37°C for 1 h. The loading control is β‐actin (dilution 1:5000, Bioss, Beijing) .A chemiluminescence kit (Millistore, WBKLS0500) was used to display the bands, and Quantity One software was used to measure the gray value of the target bands.

### 2.7 Statistical analysis

The data were analyzed using SPSS version 26.0 statistical software (IBM SPSS Inc., Armonk, NY). The results are expressed as mean ± standard deviation. Quantitative data accord with normal distribution. Measurement for PWL was analyzed by two‐way ANOVA, followed by Bonferroni post hoc test. After immunohistochemistry and western blot test, the groups were compared by one‐way ANOVA, followed by Dunnett's multiple comparison test. The cold allodynia measurement was statistically analyzed by Kruskal–Wallis rank‐sum test. Statistical significance was set at *p* < .05.

## 3 RESULTS

### 3.1 Paw‐withdrawal threshold for mechanical stimulation

**FIGURE 3:**
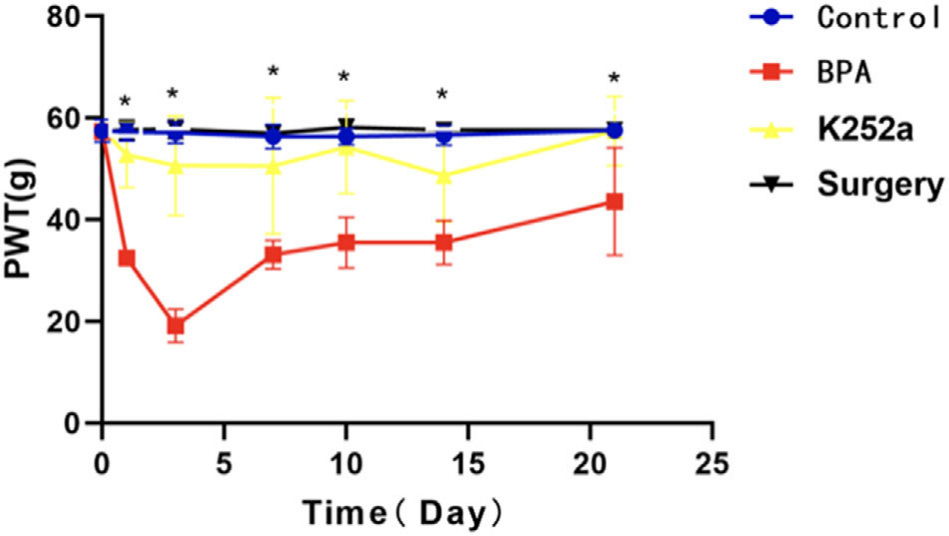
The hind paw‐withdrawal threshold of the operative side showed no difference at 0 days before operation among all groups and significantly decreased at 1, 3, 7, 10, 14, and 21 days after operation in the BPA group (*p* < .05). There was no difference at all time points between the control group and the sham operation group. After treatment with K252A, the hind paw‐withdrawal threshold was significantly higher at all postoperatively time points and consistently until 21 days postoperatively. ^*^ *p* < .05

There was no significant difference in the PWT of mechanical stimulation on the right side of the four groups before surgery (0 days) (Figure 3). The PWT for mechanical stimulation was significantly decreased on days 1, 3, 7, 10, 14, and 21 in the BPA group (*p* < .05). Compared with the BPA group at all time points after surgery, the PWT of mechanical stimulation in the K252a group was significantly higher than that in the BPA group, but showed no significant difference compared with that in the sham surgery group or the control group.

### 3.2 Cold allodynia

**FIGURE 4:**
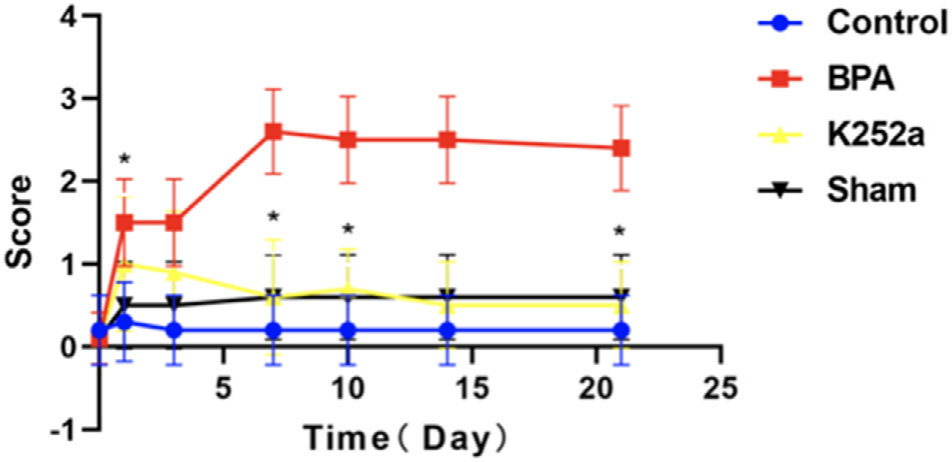
At 0 days before the operation, there was no difference in the cold allodynia scores among all group. At all time points after the operation, the score of cold allodynia in the BPA group was significantly increased, which was significantly different from that in the control group and the sham operation group. After treatment with K252a, the score of cold allodynia was significantly decreased at 1, 7, 10, and 21 days postoperatively and had no difference compared with the control group and the sham operation group. The score of cold allodynia was not significantly decreased at 3 and 14 days after treatment with K252a and had no difference compared with that of the BPA group, control group, and sham operation group. ^*^ *p* < .05

Compared with the sham surgery group and the control group, the score for cold allodynia was significantly increased from 1 to 21 days after operation in the BPA group (*p* < .05). Compared with the BPA group, the score for cold allodynia in the K252a group was significantly lower on days 1, 7, 10, and 21 than that in the control group and the sham surgery group. No difference was found between the K252a and BPA group on days 3 and 14 (Figure 4).

### 3.3 Immunohistochemistry

**FIGURE 5:**
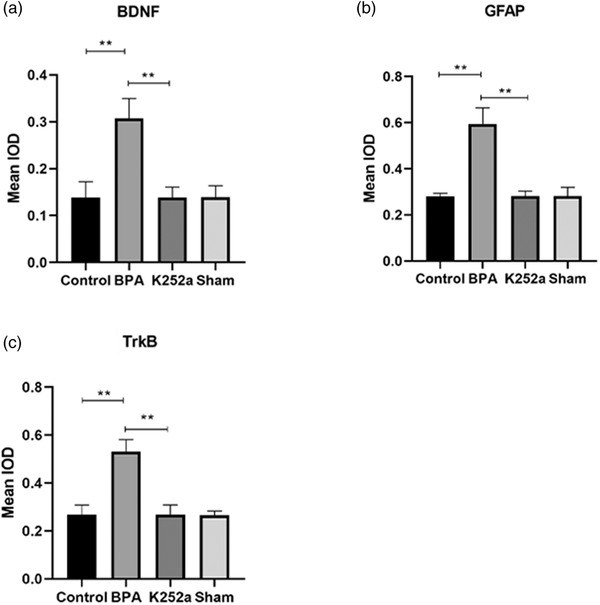
The expression of BDNF, GFAP, TrkB in the spinal cord. (a) The expression of BDNF was significantly increased in the BPA group and decreased to normal level after treatment with K252a. (b) The expression of GFAP in the spinal cord was significantly increased in the BPA group and decreased to normal level after treatment with K252a. (c) The expression of TrkB was significantly increased in the BPA group and decreased to normal level after treatment with K252a. ^**^ *p* < .01

**FIGURE 6:**
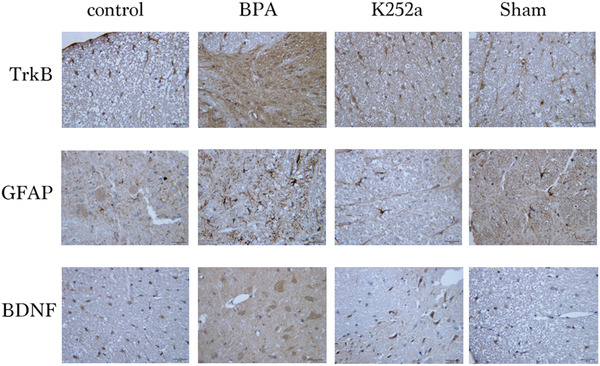
The expression of BDNF, GFAP, and TrkB at the C5–T1 dorsal horn segment in the four groups 7 days after surgery was increased compared with the sham operation and control group, but significantly decreased with K252a treatment compared with the sham operation and control group. Scale bars represent 20 µm

**FIGURE 7:**
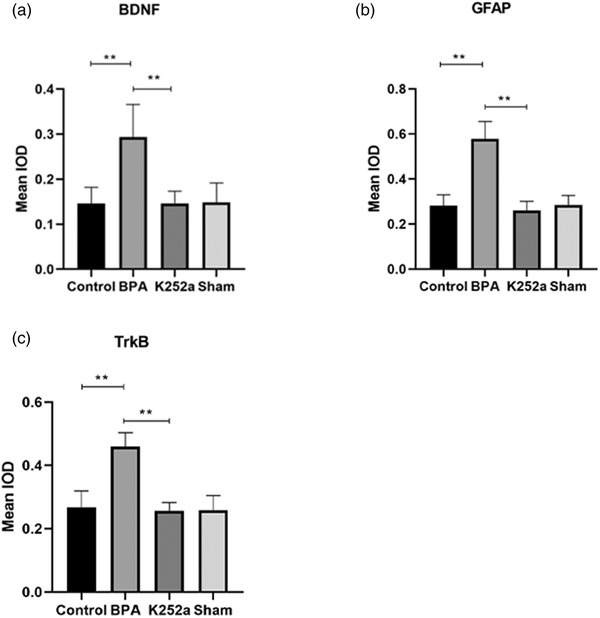
The expression of BDNF, GFAP, TrkB in the spinal cord at 21 days. (a) The expression of BDNF was significantly increased in the BPA group and decreased to normal level after treatment with K252a. (b) The expression of GFAP in the spinal cord was significantly increased in the BPA group and decreased to normal level after treatment with K252a. (c) The expression of TrkB was significantly increased in the BPA group and decreased to normal level after treatment with K252a. ^**^ *p* < .01

**FIGURE 8:**
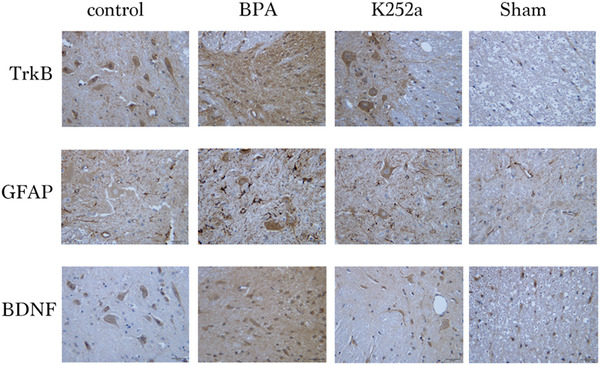
The expression of BDNF, GFAP, and TrkB at the C5–T1 dorsal horn segment in the four groups 21 days after surgery was increased compared with the sham operation and control group, but significantly decreased with K252a treatment compared with the sham operation and control group. Scale bars represent 20 µm

Compared with the sham surgery group and the control group, the expression of GFAP, BDNF, and TrkB in superficial layers of the dorsal horn of the ipsilateral C5–T1 spinal segment in the BPA group was significantly increased at 21 days after surgery. In the K252a group, the expression was significantly decreased compared to that in the BPA group (Figures 5, 6, 7, 8).

### 3.4 Western blot

**FIGURE 9:**
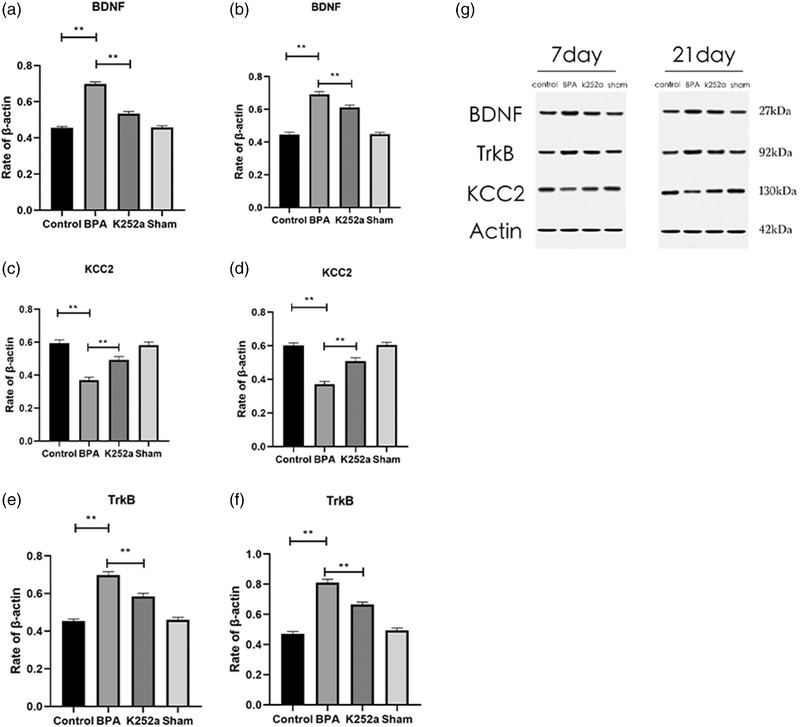
Changes in expression of BDNF, KCC2, TrkB in the spinal cord after brachial plexus avulsion and K252a treatment. (a, c, e) There was no difference in BDNF, TrkB, and KCC2 expression between the control group and the sham operation group at 7 days. The expressions of BDNF and TrkB in the BPA group increased significantly at 7 days after surgery, compared with those in the control group and the sham group, and decreased significantly at 7 days after treatment with K252a. The expression of KCC2 decreased significantly in the BPA group at 7 days, and increased significantly in the BPA group at 7 days, and increased significantly after treatment with K252a. (b, d, f) Twenty‐one days after surgery, BDNF and TrkB expression increased significantly after BPA, but decreased significantly after K252a treatment, while KCC2 expression decreased significantly after BPA and increased significantly after K252a treatment. (g) Western blot analysis of BDNF, TrkB, and KCC2 in four groups at 7 and 21 days, respectively. ^**^ *p* < .01

The expression levels of BDNF and TrkB were lower in the sham surgery group and in the control group, but was significantly increased in the BPA group, while the expression of KCC2 was significantly decreased in the sham surgery group and in the control group, and was significantly increased in the BPA group (Figure 9).

## 4 DISCUSSION

In this study, we confirmed that in a rat model of BPA, K252a treatment significantly reduced the expression of BDNF and TrkB, increased the expression of KCC2, and significantly alleviated neuropathic pain. This pathway was thus an effective target for the treatment of neuropathic pain after BPA.

Compared with other neuropathic pain models, such as sciatic nerve ligation, spared sciatic nerve injury, and spinal nerve transection, the incidence of neuropathic pain after BPA is approximately 30%–80% in clinical practice. The BPA model provides a suitable simulation for human neuropathic pain. In this study, lower trunk avulsion was used, and no self‐amputation occurred in any of the rats in the experiment. The operation time was short, and little intraoperative bleeding occurred. Root avulsion could be identified based on the dorsal root ganglion position during surgery (Yoon et al., 1994). Hyperalgesia was observed from 1st day to the 21st day after the operation. Cold allodynia scores increased from the 1st day after the operation to the 21st day and remained high.

In this study, the neuralgia markers GFAP and BDNF between the BPA group and the control group were significant on the 21st day after the operation and decreased to normal levels when K252a treatment was administered. PWT of mechanical stimulation in the K252a group showed no significant difference compared with that in the control group. Cold allodynia also decreased to close to preoperative levels on days 1, 7, 10, and 21, but there was no significant decrease on days 3 and 14. This confirmed that blocking the BDNF–TrkB pathway significantly alleviated neuropathic pain after avulsion of the brachial plexus.

In recent years, more central sensitization mechanisms have been discovered, and the nociceptive afferent fibers of the spinal dorsal horn normally receive stimuli above the nociceptive threshold and transmit pain signals. During central sensitization, the excitability of the nociceptive afferent nerve fibers in the dorsal horn increases, allowing low threshold stimulation to activate the dorsal root ganglion; this potential is transmitted through the pain pathway to initiate pain. Activation of glial cells, mainly astrocytes and microglia, is the most important trigger of central sensitization. Microglial activation is believed to be responsible for the initiation of neuropathic pain, while astrocytes are thought to be involved in the maintenance of long‐term chronic pain (Iwasaki et al., 2013). Clinically, long‐term chronic neuropathic pain is more intractable and difficult to treat. As a marker of astrocyte activation, GFAP is significantly increased in the spinal cord segment after BPA, and is an important marker of neuropathic pain (Yuan et al., 2020). As an important neurotrophic factor in the nervous system, BDNF is synthesized in dorsal root ganglion neurons, transported anterograde to superficial layers I and II of the dorsal horn of the spinal cord, and participates in pain pathway transmission through synaptic remodeling. BDNF expression is increased in the dorsal root ganglion of the ipsilateral spinal cord after nerve injury, and the BDNF‐like immunoreactive neurons are increased in the dorsal root ganglion and the superficial lamina of the dorsal horn of the spinal cord (Ha et al., 2001; Obata et al., 2003; Zhou et al., 1999). In wild mice, intrathecal injection of exogenous BDNF produced long‐lasting thermal hyperalgesia and tactile allodynia, and also increased the excitability of nociceptive afferent fibers of spinal neurons. In BDNF‐deficient mice, thermal hyperalgesia and tactile allodynia were reduced, and the excitability of primary afferent fibers were decreased (Heppenstall & Lewin, 2001). Wang et al. (2009) employed genetic methods for selective knockout of the *Trkb* gene in mice, and confirmed that TrkB was the main cause of hyperalgesia caused by heat and mechanical pain. Cao et al. (2020) proposed that the imbalance between different subtypes of TrkB is involved in abnormal signaling and hyperalgesia. In particular, inhibition of TrkB.T1, which lacks the intracellular kinase domain of the full‐length TrkB receptor and is upregulated in various CNS injury models, can reduce neuropathic pain. BDNF specifically binds to TrkB receptors to activate the process. In this study, the levels of BDNF, GFAP, and TrkB in the spinal cord of the BPA group were still significantly higher than those in the control group until day 21. After K252a treatment, the levels also decreased to near‐normal levels. The increase of BDNF, GFAP, and TrkB were almost completely reversed, suggesting that targeting this pathway is effective for long‐term chronic neuropathic pain.

The KCC2 co‐transporter, a member of the solute transporter 12 gene family, is the most important transporter in the nervous system. In the nervous system, Cl^−^ is regulated by the Na‐K‐Cl co‐transporter 1 (NKCC1) and K‐Cl co‐transporter 2 (KCC2). The Na concentration gradient is generated by Na, K‐ATPase. NKCC1 drives Cl into the cell, and KCC2 extrudes Cl into the cell. In mature neurons, the expression of KCC2 increases, while the expression of NKCC1 decreases, resulting in a low intracellular Cl concentration, leading to the depolarization to hyperpolarization GABA shift and prominent inhibition in the mature nervous system (Rivera et al., 2002). How the binding of BDNF to TrkB regulates the expression of KCC2 is not fully understood. Lee‐Hotta et al. (2019) speculated that it might regulate the expression of KCC2 through the PLCγ1 and SHC pathways. BDNF binding to TrkB leads to autophosphorylation of TrkB intracellular tyrosine residues and activates the phospholipase C–calmodulin‐dependent protein kinase pathway, the phosphoinositol kinase 3 pathway, and the RAS/MAPK pathway. Thereby, it regulates a decrease in KCC2 expression, resulting in a decrease in Cl^−^ extrusion and an increase in the Cl^−^ concentration in neurons. This leads to hyperpolarization of the Cl^−^ gradient, which may subsequently lead to the shift in GABA signaling from inhibition to excitation. Coull et al. (Coull et al., 2005) found that the concentration of anions in neurons correlated positively with the initiation and development of neuralgia, that KCC2, as the main Cl^−^ extruder in the nervous system, plays a key role in changes in the neuronal excitation state. In this study, western blotting results showed that BDNF and TrkB levels remained high in the BPA group at 7 and 21 days postoperatively, while those in the K252a group significantly decreased by 7 and 21 days postoperatively. The expression level of KCC2 in the control group was maintained at a high level, while that in the BPA group was significantly decreased at both time points, which was significantly different from that in the control group. However, with K252a treatment, KCC2 increased significantly, confirming that KCC2 plays an important role in the development of neuropathic pain. We speculate that the downregulation of KCC2 may lead to transformation of GABA from inhibitory to excitatory, causing neuropathic pain.

This study had some limitations. The sample size of the study was relatively small, and the experimental results may not reflect the true effect. This study did not compare the effects of other neuralgia pathways, or of the combined effects of various pathways, such as CCL2–CCR2, TNF–p55, and CXCL12–CXCR4. Whether these pathways represent different components of or interact in pain transmission need to be investigated in previous studies. Additionally, there is no control group of animals treated with K‐252a, which evaluated whether the effects on TrkB inhibition are specific for the neuropathic condition or not.

In conclusion, no previous study had shown that the BDNF–TrkB pathway regulates the expression of KCC2, plays an important role in the development of neuropathic pain after BPA, and is involved in the initiation and maintenance of neuropathic pain. Our findings suggest that blocking the BDNF–TrkB–KCC2 pathway may be an effective therapeutic target for neuropathic pain after BPA.

## FUNDING INFORMATION

This research received no specific grant from any funding agency in the public, commercial, or not‐for‐profit sectors.

## CONFLICT OF INTEREST

The authors declare that there is no conflict of interest.

## Data Availability

All data included in this study are available upon request by contact with the corresponding author.
